# Follicular Fluid Zinc Level and Oocyte Maturity and Embryo Quality in Women with Polycystic Ovary Syndrome

**DOI:** 10.22074/IJFS.2021.135426.1006

**Published:** 2021-06-22

**Authors:** Sima Janati, Mohammad Amin Behmanesh, Hosein Najafzadehvarzi, Zahra Akhundzade, Seyedeh Mahsa Poormoosavi

**Affiliations:** 1 Department of Obstetrics and Gynecology, School of Medicine, Research and Clinical Center for Infertility, Dezful University of Medical Sciences, Dezful, Iran; 2 Department of Histology, School of Medicine, Dezful University of Medical Sciences, Dezful, Iran; 3 Department of Pharmacology, Faculty of Medicine, Babol University of Medical Sciences, Babol, Iran; 4 School of Medicine, Dezful University of Medical Sciences, Dezful, Iran; 5 Department of Histology, School of Medicine, Research and Clinical Center for Infertility, Dezful University of Medical Sciences, Dezful, Iran

**Keywords:** Embryo, Oocyte, Polycystic Ovary Syndrome, Zinc

## Abstract

**Background:**

Polycystic ovary syndrome (PCOS) is considered to be one of the most common endocrine disorders in women of reproductive age. Zinc, a vital trace element in the body, plays a key role in maintaining health, especially due to its antioxidant role. On the other hand, lack of antioxidants and oxidative stress can adversely affect oocytes quality and consequently fertility rate. The available studies that report the effect of follicular fluid (FF) zinc in terms of the number and quality of the oocytes in infertile women with PCOS, are few and not consistent. We decided to investigate this issue.

**Materials and Methods:**

In this cross-sectional study, from the women with PCOS referring to Omolbanin Hospital, Dezful, Iran (February to December 2019), a total of 90 samples (follicular fluid, oocytes, and embryos) were collected from those who had undergone in vitro fertilization (IVF). To measure zinc level in follicular fluid, high performance liquid chromatograpy (HPLC) was utilized. Also, oocytes maturity and embryos quality evaluation was performed using inverted optical microscopy. One-way ANOVA and Fisher’s least significant difference (LSD) were used for data analysis.

**Results:**

The amount of FF zinc was not associated with any significant differences in the number of oocytes and metaphase I (MI) and germinal vesicle (GV) oocytes, but a significant decrease was observed in the number of metaphase II (MII) oocytes at zinc values less than 35 µg/dL. The FF zinc levels less than 35 µg/dL were also significantly associated with decreased embryo quality

**Conclusion:**

A significant relationship was found between the level of FF zinc and the quality and the number of oocytes taken from the ovaries of infertile patients with PCOS history who were candidates for IVF treatment as well as the number of high quality embryos.

## Introduction

As one of the most common endocrinopathies, polycystic ovary syndrome (PCOS) is reported to affect 6-10% of reproductive-age women (1, 2); its prevalence rate is 5-10% in the general population and almost 30% among overweight women (3). This including hirsutism, amenorrhea, hyperinsulinemia, obesity, and hyperandrogenism. PCOS attributes to 3/4 of the ovulatory infertility cases (4). A high risk of endometrial and ovarian cancer has been shown in PCOS cases (5). 

The oocytes obtained from PCOS patients who endure *in vitro* fertilization (IVF) often have low quality, leading to high rates of cancelation and low fertilization (6). Abnormal increased levels of androgen and/or insulin seem to be the main underlying pathophysiological mechanism for PCOS. Obesity worsens the condition of PCOS patients (7). Nonetheless, the complexity of PCOS, and how PCOS affects the oocytes development, are not fully understood and need further studies. The follicular fluid (FF) is produced by granulosa and theca cells in the growing antral follicles (8). It provides a micro-environment for the oocytes development and contains several factors including steroids, polysaccharides, proteins, antioxidant enzymes and trace elements which modulate the oocyte developmental capacity and ovulation. The FF also serves as a medium for the communication between follicular cells and oocytes during follicular development. The FF composition may reflect changes in ovarian cells secretory processes and changes in the plasma components due to pathological conditions (9).

The trace elements in human tissues are essential for cell growth, maturity, and physiological functions. For more than 300 proteins, enzymes, and transcriptional factors activities, Zinc is a main trace element present in the oocytes and FF(10) making it a structural, catalytic and regulatory ion (11). Hence, for maintaining homeostatic responses in the body including oxidative stress and several biological functions such as immune efficiency, zinc is crucial. zinc paucity in females may cause issues like reduced synthesis/ secretion of follicle stimulating hormone (FSH) and luteinizing hormone (LH), estrous cycle disruption, extended gestation period, ovarian insufficiency, frequent abortion, teratogenicity, stillbirths, pre-eclampsia, toxemia, complexity in parturition, and lower infant birth weights (12).

Oocytes indicate the zinc transporters, metal regulatory transcription factors and metallothioneins. This highlights a substantial role for zinc, especially with possible association with the genome stability during early embryonic development (13). It is well known that IVF is one of the critical treatments for infertility, but the clinical pregnancy rate of IVF is affected by multiple factors including zinc level (14). 

To date, however, there are only a few papers determining the trace elements levels in FF and serum of PCOS patients who undergo IVF, and the data are not consistent. Therefore, we aimed to measure zinc levels in the FF of PCOS patients who underwent IVF and find out the correlation between zinc level and oocytes and embryo quality

## Materials and Methods

This cross-sectional research studied 90 PCOS women (20-45 years old) who were selected from patients referring to the Omolbanin Infertility Center, Ganjavian Hospital, Dezful, Iran (October 2018-September 2019), using simple random sampling. The nominated subjects received assisted reproductive technology (ART) for infertility. The data was obtained via questions that investigated the age of men and women and the causes or duration of infertility, taking supplements comprising zinc in the past two months, body weight and height, and body mass index (in women).This research was approved by Dezful University Ethics Committee (IR.DUMS.REC.1397.005). Prior to initiation, the participants signed the informed consent forms. Controlled ovarian stimulation was performed for all the patients using the antagonist protocol (15). The participants received oral contraceptive pills (Ocp LD) in low-dose (0.3 mg norgestrel and 30 μg ethinyl estradiol, Aburaihan Pharmaceutical Co., Iran) which began on the 2^nd^ day of the cycle then discontinued till menstruation occurred. Once menses began, gonadotropin stimulation was started by Gonal-F (Gonal- F, Serono, Italy) from the 2^nd^ day of the menstrual cycle. The gonadotropin starting dose was 150-300 mIU/d, based on the patient’s body weight and age. The patients were monitored on day 7 of stimulation and dose of gonadotropin was adjusted based on the serum estradiol (E2) concentrations and ovarian response as observed by ultrasound. Once the leading follicles reached 14 mm in diameter, Cetrorelix (Merck-Serono, Germany) was added subcutaneously (0.25 mg) and continued every day till the human chorionic gonadotropin (hCG) administration day. In all patients, 10,000 IU of hCG (Pregnyl, Daropakhsh, Iran) was administered intramuscularly (IM) when at least three follicles reached more than 18 mm in diameter. Then, 36 hours after hCG, the ovum was picked up in an ultrasound-guided manner. The puncturing was performed to remove oocytes and the FF for the aspiration by catheter. To avoid sample contamination from blood first tube of the FF was used. The samples were transferred to the test tubes. The oocytes were washed using G-MOPS medium (Vitrolife-Sweden), and then, incubated for two hours at 37ºC with 6% CO_2_ . After oocytes removal, the FF cells were pellet-ed by centrifugation at 3,000 rpm for 10 minutes. Then, blood-free supernatant was aliquoted and stored at -70°C until further evaluation (16). Using an inverted microscope (Olympus, Japan), the oocytes were classified into three categories. The categories were as follows: metaphase II (MII) (presence of the first polar body), metaphase I (MI) (absence of the first polar body and germinal vesicle breakdown), and germinal vesicle (GV) and degenerated oocytes (17). The FF sample (1 ml) was taken from each patient. Subsequently, the oocytes were inseminated. Pro-nucleolus (PN) score was noted 16-18 hours following insemination. The embryo quality (A, B or C) was evaluated before embryo transfer according to the reference (18).

Zn assays were performed via a colorimetric method using spectrophotometry at 560 nm, and dye (2,5-bromo-2-pyridylazo)-5- (N-propyl-N-sulpho-propylamino) phenol at a slightly acidic pH value. Trichloracetic acid was used for protein precipitation. Salicylaldoxime and dimethylglyoxime were included in the assay for zinc extraction and transition metal chelator, respectivel

### Statistical analysis

To assess the differences among the sample groups to be significant we used Fisher’s least significant difference (LSD) post-hoc test. Also, to do a comparison among the variances of the three sample groups, one-way analysis of variance (ANOVA) was conducted. To do the analysis, SPSS 22 (SPSS Inc., Chicago, Ill., USA) was used while the significance level was set at less than 0.05.

## Results

### Patients and oocytes

Totally, 740 oocyte samples were collected from 90 women enrolled in the current research. The mean oocyte count was 8.2. The mean counts of MII, MI, and GV along with degenerated oocytes were approximated at 7.67, 1.1, 0.94, respectively.

**Table 1:** Comparing the average number of oocytes considering different groups of variables


Variables	Oocyte number	MII	MI	GV, Degenerated

Women age (Y)				
25-30	12.12 ± 4.32^b^	4.17 ± 4.21^a^	5.46 ± 2.88^a^	2.36 ± 3.17^b^
30-35	11.35 ± 3.74^b^	4.36 ± 4.21^a^	5.82 ± 4.42^a^	2.7 ± 3.41^b^
35-40	11.27 ± 2.99^b^	2.71 ± 4.51^b^	5.31 ± 5.38^a^	3.16 ± 3.91^b^
40-45	12.64 ± 4.51^b^	2.4 ± 2.44 ^b^	4.99 ± 5.12^a^	6.1 ± 4.27^a^
≥45	12.21 ± 1.83^b^	2.01 ± 4.12^b^	5.01 ± 3.49^a^	5.4 ± 4.28^a^
Women BMI (kg/m^2^)				
<25	11.27 ± 3.51^a^	5.34 ± 2.68^a^	4.77 ± 3.42^a^	5.27 ± 2.37^a^
25-30	10.56 ± 3.98^a^	3.27 ± 3.81^b^	4.99 ± 48^a^	2.74 ± 4.71^b^
≥30	7.1 ± 4.11^b^	1.24 ± 4.52^c^	4.39 ± 4.74^a^	2.41 ± 2.73^b^
Cause of infertility				
Male factor	12.77 ± 6.42^a^	6.33 ± 4.97^a^	5.47 ± 3.71^a^	1.18 ± 3.07^a^
Female factor	8.21 ± 4.91^b^	2.27 ± 3.42^b^	4.77 ± 3.23^a^	2.1 ± 2.72^a^
Both	8.28 ± 3.91^b^	2.97 ± 3.342^b^	4.98 ± 4.18^a^	1.77 ± 3.71^a^
Infertility duration (Y)				
≤5	8.14 ± 3.61^b^	2.55 ± 5.12^a^	4.27 ± 3.91^a^	3.47 ± 2.35^a^
>5	11.44 ± 3.41^a^	2.98 ± 3.81^a^	5.32 ± 4.01^a^	3.22 ± 3.34^a^


Data are presented as mean + SD. MII; Metaphase II, MI; Metaphase I, GV; Germinal vesicle, BMI; Body mass index, and^ a^ , ^b^, ^c^ ; Designate significant differences (P≤0.05).

### Comparison of the studied variables in terms of oocyte maturity

Based on the results, the mean count of MII oocytes was significantly higher in the subjects aged less than 35 years old (P≤0.05), as well as those with lower body mass index (BMI) and male infertility factor (P≤0.05). However, in terms of infertility duration, no significant difference was observed in the mean count of MII oocytes among the study groups (P>0.05, Table 1).

### The levels of zinc in the follicular fluid and its association with oocyte maturity

Between the zinc level in the FF and the counts of oocytes, MI oocytes and GV oocytes, no significant association was detected (P>0.05). The mean count of the MII oocytes in women with zinc levels less than 35 µg/ml, was significantly lower (P≤0.05). For MII oocytes, the highest mean (6.25 ± 3.6) and lowest mean (3.45 ± 3.6) was detected for the zinc levels of 35-45 µg/ml and 25-35 µg/ml in the FF, respectively (Table 2). 

### Patients and embryos

To tally, 450 embryos were collected from 82 women. There were 8 cases with no embryo. The mean number of embryos was 5.48 ± 3.98 with the range of 1 to 16. Most of the embryos (124 cases) were qualified as A, 193 cases as B and 133 cases as C.

### Comparison of the studied variables in terms of embryo quality

Those with a BMI less than 25, age<35 years, and infertility duration of <5 years and couples with male infertility had embryos with significantly higher quality (P≤0.05). For cases with man’s age>45 years, woman’s BMI greater than 30 and woman’s age >35 years, a greater percentage of obtained embryos showed significant C quality (P≤0.05, Table 3).

**Table 2:** Comparing the average distribution of oocytes among different levels of zinc in FF


Zinc(µg/dl)	Oocyte number	MII	MI	GV,Degenerated

15-25	11.74 ± 3.65^a^	3.95 ± 3.49^b^	6.35 ± 4.42^a^	1.28 ± 5.21^a^
25-35	12.21 ± 3.27^a^	3.45 ± 3.61^b^	7.41 ± 3.65^a^	2.96 ± 4.21^a^
35-45	13.49 ± 4.51^a^	6.25 ± 3.61^a^	6.28 ± 3.72^a^	1.55 ± 4.42^a^
45-55	13 ± 4.91^a^	6.1 ± 2.51^a^	6.1 ± 3.21^a^	1.3 ± 3.71^a^


Data are presented as mean + SD. FF; Follicular fluid, MII; Metaphase II, MI; Metaphase I, GV; Germinal vesicle, and ^a^ , ^b^, ^c^ ; Designate significant differences (P≤0.05).

### Zinc levels in the follicular fluid and its association with embryo quality

The mean count of embryos had grade A quality which was significantly lower in the women with zinc levels less than 45 µg/ml (P≤0.05). The mean count of embryos with B quality was significantly lower in women with zinc levels <35 µg/ml (P≤0.05, Table 4).

**Table 3:** : Comparing the average number of embryos with A, B or C quality among different variable groups


Variables	Grade A	Grade B	Grade C

Women age (Y)			
25-30	8.34 ± 4.22^a^	7.66 ± 3.88^a^	1.41 ± 1.91^b^
30-35	8.45 ± 3.15^a^	6.97 ± 4.72^a^	1.36 ± 4.21^b^
35-40	4.21 ± 2.51^b^	3.31 ± 5.38^b^	3.71 ± 4.51^a^
40-45	2.25 ± 3.52^c^	3.99 ± 4.52^b^	3.42 ± 2.44^a^
≥45	2.3 ± 3.83^c^	1.71 ± 2.45^c^	4.09 ± 4.12^a^
Men age (Y)			
25-30	7.48 ± 4.22^a^	7.02 ± 4.28^a^	1.98 ± 3.31^b^
30-35	8.45 ± 3.15^a^	6.68 ± 5.82^a^	1.48 ± 3.47^b^
35-40	4.71 ± 5.26^b^	6.14 ± 5.38^a^	3.55 ± 4.28^b^
40-45	4.3 ± 4.28^b^	2.49 ± 4.51^b^	3.97 ± 2.29^a^
≥45	1.4 ± 6.43^c^	2.55 ± 4.28^b^	4.17 ± 3.71^a^
Women BMI (kg/m^2^)			
<25	12.24 ± 4.31^a^	7.09 ± 4.74^a^	1.87 ± 3.31^b^
25-30	8.55 ± 4.61^b^	6.33 ± 4.81^a^	1.48 ± 3.25^b^
≥30	7.71 ± 3.15^b^	6.72 ± 3.22^a^	3.55 ± 4.32^a^
Cause of infertility			
Male factor	7.24 ± 3.71^a^	7.23 ± 2.91^a^	6.88 ± 5.34^a^
Female factor	5.37 ± 3.41^b^	5.27 ± 6.11^b^	8.78 ± 2.91^a^
Both	3.84 ± 3.71^a^	3.05 ± 4.42^a^	2.84 ± 5.51^a^
Infertility duration (Y)			
≤5	12.37 ± 4.52^a^	7.23 ± 5.61^a^	3.87 ± 3.71^a^
>5	6.58 ± 3.92^b^	6.98 ± 3.31^a^	4.32 ± 2.36^a^


Data are presented as mean + SD. BMI; Body mass index, and ^a^ , ^b^, ^c^ ; Designate significant differences (P≤0.05).

**Table 4:** Comparison of the average number of embryos with A, B or C quality among different levels of zinc in FF


Zimc (µg/dl)	Grade A	Grade B	Grade C

15-25	3.58 ± 3.66^b^	2.64 ± 5.25^b^	2.3 ± 2.92^b^
25-35	4.23 ± 3.52^b^	3.18 ± 3.42^b^	1.54 ± 3.16^b^
35-45	3.44 ± 3.66^b^	6.35 ± 4.21^a^	2.1 ± 4.12^b^
45-55	7.25 ± 3.45^a^	6.14 ± 4.12^a^	4.5 ± 4.11^a^


Data are presented as mean + SD. FF; Follicular fluid, and ^a^ , ^b^, ^c^ ; Designate significant differences (P≤0.05).

## Discussion

PCOS is one of the most prevalent endocrine-metabolic ailments. It can be specified as a combination of anovulation (oligomenorrhea, infertility, and dysfunctional uterine bleeding) and hyperandrogenism (acneand hirsutism) along with polycystic ovaries. The effect of FF on the oocytes and embryos development was ratified; lack of several elements and nutrients may lead to a reduction in the possibility of successful natural fertility (19). In the current study, we determined the zinc level in the FF and assessed embryo quality in PCOS patients who underwent IVF.

Zn is present in all cells of the body, taking part in more than 200 enzymes formation. In fact, it performs important roles in proper function of different enzymes. According to the studies, zinc deficiency in women can lead to abnormalities in the production and secretion of FSH and LH, abnormal ovarian differentiation, recurrent miscarriage, etc. (20). In 2017 Sun et al. (21) observed a positive correlation between zinc in the FF and the number of oocytes in the patients undergoing IVF. They stated that low levels of zinc decrease the number of oocytes and their quality, which is in accordance with the present study results.

According to this research, there is a positive association between decreased oxidative stress and increased oocyte maturation in the PCOS and infertile women. It can be said that zinc has antioxidant properties and reduces oxidative stress in patients with PCOS during IVF. zinc deficiency significantly increases apoptosis induced by the cytokines, and oxidative stress in somatic cells (22). zinc deficiency-induced apoptosis can inhibit cumulus cells proliferation. The cumulus cells play important roles in oocytes maturation and they are needed for the cytoplasmic maturation and growth because they synthesize glutathione (GSH) and transmit it to the oocytes. Thus, poor growth and development of cumulus cells negatively affect oocyte maturation and quality (23). If zinc supplementation is done and its level reaches normal values, the number and quality of the oocytes will improve. Their results are consistent with the present study outcome. According to the findings of the present study, a reduction in zinc will decrease the number of better quality embryos. The findings of recent studies indicate that zinc is very important for the meiotic cell cycle regulation and ovulation (24). Nevertheless, Zn’s role in promoting oocyte quality and growth potential, is not known yet. Research suggests that zinc deficiency in women just prior to ovulation, disrupts the epigenetic programming of the oocyte, including a decrease in DNA and histone protein methylation. These epigenetic deficiencies, along with meiotic defects, compromise fertilization and the embryo growth. Dietary deficiency of zinc reduces the potential for oocyte growth (25). The major part of the embryo cytoplasm originates from the oocyte. In fact, this is the oocyte that provides the required components to support fetal growth, such as mRNA and protein, to activate the fetal genome and maintain its growth. The quality of the oocyte largely determines the achievement of fertilization and the early development of the fetus. The ovary environment can determine the oocyte quality, but the mechanisms of optimal oocyte growth and maturation are not fully discovered yet. Diet and environment can impair the reproductive performance, including oogenesis at different stages. Recent findings have shown that zinc is an important factor in maintaining meiosis arrest before puberty (26). zinc is also required to complete meiosis I during laboratory puberty. Studies have shown that acute zinc deficiency reduces the oocytes quality (27). This finding is consistent with the results of the present study. zinc is also required for the synthesis of vitamin A reductase and zinc deficiency may decrease serum vitamin A levels, possibly leading to oocyte failure (28).

## Conclusion

According to the obtained results, there is a positive correlation between the level of FF zinc and the quality and maturation of oocytes taken from ovaries of infertile subjects with PCOS history. Also, among our participants, the embryos of subjects who underwent IVF and had higher FF zinc levels, had higher quality. There is not sufficient knowledge about the exact effect of zinc on the oocyte and embryonic quality, thus, further investigations on higher numbers of patients for further validation, are recommended.
